# The Role of S1P and the Related Signaling Pathway in the Development of Tissue Fibrosis

**DOI:** 10.3389/fphar.2018.01504

**Published:** 2019-01-08

**Authors:** Erjin Wang, Xingxuan He, Ming Zeng

**Affiliations:** ^1^Department of Health Toxicology, Xiangya School of Public Health, Central South University, Changsha, China; ^2^Department of Human Genetics and Genomic Sciences, Icahn Institute for Genomics and Multiscale Biology, Icahn School of Medicine at Mount Sinai, New York, NY, United States

**Keywords:** sphingosine 1-phosphate, tissue fibrosis, SphK, S1PRs, clinical treatment

## Abstract

Tissue fibrosis, including pulmonary fibrosis, hepatic fibrosis, and cardiac fibrosis, is an important stage in the development of many diseases. It can lead to structural damage and dysfunction and even severe carcinogenesis or death. There is currently no effective method for the treatment of fibrosis. At present, the molecular mechanism of tissue fibrosis has not yet been fully elucidated, but many studies have demonstrated that it is involved in conveying the complex messages between fibroblasts and various cytokines. Sphingosine 1-phosphate (S1P) is a naturally bioactive sphingolipid. S1P and the related signaling pathways are important intracellular metabolic pathways involved in many life activities, including cell proliferation, differentiation, apoptosis, and cellular signal transduction. Increasing evidence suggests that S1P and its signaling pathways play an important role in the development of tissue fibrosis; however, the mechanisms of these effects have not yet been fully elucidated, and even the role of S1P and its signaling pathways are still controversial. This article focuses on the role of S1P and the related signaling pathways in the development of fibrosis of lung, liver, heart, and other tissues, with emphasis on the application of inhibitors of some of molecules in the pathway in clinical treatment of fibrosis diseases.

## Introduction

### Tissue Fibrosis

Tissue fibrosis is the formation of excess fibrous connective tissue in a reparative or reactive process. It is usually observed after tissue injury in the lung, liver, heart, skin, eyes, and other organs. Some fibrotic diseases, such as idiopathic pulmonary fibrosis (IPF) and systemic sclerosis (SSc), can even lead to systemic progressive fibrosis and death (Varga and Abraham, 2007; Ley et al., 2011).

The development of tissue fibrosis is generally sustained and progressive. At present, almost all kinds of fibrosis have no effective treatment, and the existing treatment can only improve the quality of life or prolong the life of patients to a limited extent. Therefore, it is urgent to find new precise signaling pathways or molecules participating in tissue fibrosis, and to explore new therapeutic strategies for tissue fibrosis in order to control the progressive development of the disease and even cure the disease. The S1P (sphingosine 1-phosphate) signaling pathway has become a hot spot in the research of fibrosis mechanisms in recent years because it is involved in the development of fibrosis. In many *in vitro* or *in vivo* experiments, inhibitors of this signaling pathway have been proven to effectively inhibit the development of multiple types of fibrosis, which provides a new thought for the treatment of fibrosis and the related diseases.

### Signaling Pathways Mediated by S1P

In recent years, the role of lipid in intercellular signal transduction has attracted increasing attention. The sphingomyelin (SM) signaling pathway is one of the main lipids of interest, which is involved in many activities of cells and organs, including cell survival, proliferation, differentiation, and diseases, such as cancer, infection, neurodegenerative disorders, and fibrosis.

**FIGURE 1:**
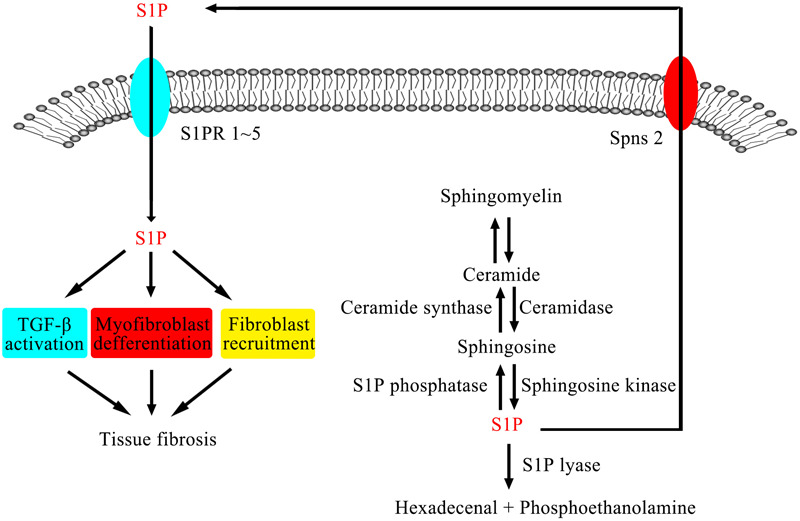
SMase catalyze SM to produce Cer, while CDase catalyze Cer to produce Sph, and S1P can be generated by SphKs. After it is generated, S1P is secreted outside the cell by S1P transporter or degraded as ethanolamine phosphate and hexadecanal by S1PL rather than accumulating in cells under normal circumstances. S1PRs are a kind of G protein coupled receptors. Through binding to different receptors, S1P regulates many physiological or pathological processes.

The metabolic pathway of the SM signaling pathway is shown in Figure 1. In several mammalian cells, sphingomyelinases (SMase) catalyze SM to produce ceramide (Cer), while ceramidases (CDase) catalyze Cer to produce sphingosine (Sph), and S1P can be generated by two isoforms of sphingosine kinases (SphKs), sphingosine kinase 1 (SphK1) and sphingosine kinase 2 (SphK2). At the same time, S1P can also be transformed into S1P phosphatase (S1PP) by intracellular Sph (Maceyka and Spiegel, 2014). After it is generated, S1P is secreted outside the cell by S1P transporter or degraded as ethanolamine phosphate and hexadecanal by S1P lyase (S1PL) rather than accumulating in cells under normal circumstances (Serra and Saba, 2010). S1P receptors (S1PRs, lysophospholipid receptors) are a kind of G protein coupled receptors, which have five different subtypes: S1P_1-5_. S1PRs are located in different tissues. For example, S1P_1_, S1P_2_, and S1P_3_ are widely expressed in multiple tissues; however, the expression of S1P_4_ is limited to lymphatic and hematopoietic tissues, and S1P_5_ is expressed in the central nervous system. Through binding to different receptors, S1P regulates many physiological or pathological processes (Xiu et al., 2015). At the same time, the expression of S1PRs is also regulated by S1P (Sanchez and Hla, 2004). S1P, as a downstream product of SM pathway, plays an important role in many life activities. This article focuses on the role of S1P and its signaling pathway, that is, the relationship between S1P, SphK, S1PRs, and S1PL, in fibrosis of the lung, liver, heart, and other tissues (summarized in Table 1) and the value of their clinical application.

**Table 1:** Overview of the role of S1P and the related signaling pathway in different types of fibrosis models.

Organ/Tissue	Model	Receptor(s)	SIP	Other sphingolipid or enzyme	Effects	Reference
Lung	IPF patients	–	↑	S1PR2↑, S1PR3↑, S1PL↑, SphK1↑, SphK2–	α-SMA ↑, activation Smad3 and RhoA-GTP, oxidative stress↑, TGF-β1↑, FN↑	Milara et al., 2012; Huang et al., 2013a, 2015; Huang and Natarajan, 2015
	Normal human lung fibroblasts	1–5	?	?	α-SMA –, ECM synthesis↑, activating PI3K/Akt and ERK1/2 signaling	Sobel et al., 2013
	Human lung fibroblasts WI-38	1, 2, 3	↑	SphK1↑	α-SMA ↑, FN↑	Kono et al., 2007
						Urata et al., 2005
	Mice	2, 3	↑	SphK1↑, SphK2–, S1PL↑	α-SMA ↑, FN↑	Kono et al., 2007; Milara et al., 2012; Huang et al., 2013a, 2015; Huang and Natarajan, 2015
Liver	Hepatic myofibroblast	1, 2, 3	↑	SphK1↑, SphK2–	α-SMA ↑, Col I↑, Col III↑	Li et al., 2011
	HSCs	1, 2	↑	SphK1↑	α-SMA ↑, Col I↑, Col III↑	Spiegel and Milstien, 2007; Brunati et al., 2008
	LX-2 cell line	1, 2, 3	?	SphK1↑	α-SMA ↑, Col I↑, Col III↑, HYP↑, FN↑	Liu et al., 2011; Wang et al., 2015
	Liver cancer patients	1, 2, 3	–	SphK1↑, SphK2–, SPNS2↑	α-SMA ↑	Sato et al., 2016
	Mice	1, 2, 3	↑/–	SphK1↑, SphK2–, S1PL–/↓|, ASMase↑	α-SMA ↑, FN↑, TGF-β↑, Ang1↑, CD31↑, Col I↑, Col III↑, cell migration↑, HuR mRN↑a	Li et al., 2009a; Kong et al., 2014; Gonzandez-Fernandez et al., 2017
						Ikeda et al., 2009; Li et al., 2009b; Yang et al., 2012, 2013; Ding et al., 2016; Chang et al., 2017; King et al., 2017; Wang et al., 2017
Heart	Cardiac fibroblasts	1, 2, 3	↑	SphK1↑, Cer↓, Sph↑	Col ↑, IL-6↑, MMP-9↑, α-SM↑a, TGF-β↑	Kacimi et al., 2007; Lowe et al., 2009; Benamer et al., 2011
	Mice	1	?	?	ANP↑, BNP↑, Col I↑, α-SM↑a, CD31↑, ERK↑, IL-6↑	Ohkura et al., 2017
Skeletal muscle	Murine C2C12 myoblasts	2, 3, 4	↑	SphK1↑, SphK2–, S1PR3↑, Cer↑, Sph–	α-SMA ↑	Cencetti et al., 2010
Kidney	Mice	?	↑	SphK1↑	ECM↑, Col IV↑, FN↑, Mcp-1↑	Wang et al., 2005; Yaghobian et al., 2016
Gluteal muscle	Gluteal muscle contracture (GMC)	?	↑	?	α-SMA ↑, cell Proliferation↑, TGF-β↑, PAI1↑	Zhao et al., 2011
Eye	Primary human retinal pigmented epithelial (RPE) cells	1, 2, 3, 5	?	SphK1↑, SphK2↑	α-SMA ↑, PAI1↑, HSP47↑	Swaney et al., 2008

*↑, upgrade; ↓, downgrade; –, unchanged; ?, unmeasured.*

In recent years, several studies have shown that some small molecular lipids, such as lysophosphatidic acid (LPA) (Zhao and Natarajan, 2013) and S1P (Nagahashi et al., 2014), are upgrade in the occurrence of diseases, such as cancer, inflammation, and tissue fibrosis. S1P is a naturally bioactive sphingolipid. S1P in plasma is mainly derived from red blood cells and activated platelets, which lack S1PL (Yatomi et al., 2001), or are phosphorylated by SphK1 (Venkataraman et al., 2006). A variety of cells, including red blood cells, platelets, macrophages, mast cells, and endothelial cells, have been confirmed to be involved in the synthesis of S1P and secret S1P into the blood (Thuy et al., 2014). S1P in blood is usually not dissociated but is closely bound up with albumin and high-density lipoprotein (HDLP) in plasma (Wilkerson et al., 2012). A study has shown that the half-life of S1P in mouse plasma is only 15 min, which indicates that it has a rapid enzymatic reaction in the plasma, and S1PP and/or S1PL plays an important role in the reaction (Nagahashi et al., 2016). S1P maintains a high level (1–2 μM) in the blood, implying that there is an efficient S1P production source in cells to maintain a high level of S1P (Salous et al., 2013). However, the concentrations of S1P in lymphatic tissues are relatively low (<0.2 μM). The concentration gradient between blood and lymph may be related to the regulation of the immune cell transportation between the body circulation and the lymphoid tissue (Donoviel et al., 2015), and the difference of concentration may be a key factor in the migration of fibroblasts and cancer cells (Cyster and Schwab, 2012).

In recent decades, functions of S1P have been determined by several studies under normal physiological conditions. In general, there are two main functions. Firstly, S1P acts as an intracellular second messenger involved in regulating cell proliferation and inhibiting apoptosis by binding to the receptor S1PRs (Pyne et al., 2016), whose function will be described in detail later in this article. Secondly, it becomes a regulator after translating out of cells regulating the cytoskeleton remodeling and cell migration, angiogenesis, and vessel maturation, the immune and lymphatic cell transport, and inflammation and malignant transformation (Hla et al., 2001; Christoffersen et al., 2011; Maceyka et al., 2012; Blaho et al., 2015; Galvani et al., 2015; Park and Im, 2017).

Sphingosine 1-phosphate is widely assumed to play a protective role in the vascular system and tissues at the physiological level, while the abnormal activation of the S1P associated signaling pathway usually leads to fibrosis, cancer, and vascular dysfunction (Delgado et al., 2012; Bhavanam et al., 2014), and S1P plays an important role in promoting fibrosis, apoptosis, inflammation, and migration in these cases (Spiegel and Milstien, 2003).

Recent studies have shown that S1P plays a crucial role in tissue fibrosis (Pyne et al., 2013; Schwalm et al., 2013), and the tissue and/or plasma concentrations of S1P are associated with a variety of fibrosis factors, including transforming growth factor β (TGF-β), platelet-derived growth factor (PDGF), and connective tissue growth factor (CTGF) (Sauer et al., 2004). Both intracellular and extracellular S1P are reported to be involved in fibrosis activities. The increase of S1P in peripheral blood is seen in a variety of fibrosis-related diseases, and the use of anti-S1P antibody to neutralize S1P can effectively reduce TGF-β-mediated collagen production (Lowe et al., 2009). In addition, a study indicates that intracellular and extracellular S1P affect cell growth and survival through different pathways (Olivera et al., 2003). Studies have also indicated that S1P-mediated autophagy may be one of the pathways in the process of fibrosis (Tatler and Jenkins, 2015), which is further described later in this article. In general, studies about the role of S1P in fibrosis have mainly focused on the effect of the secretion of key cytokines and the accumulation of extracellular matrix (ECM) after apoptosis, external chemical stimulation or hereditary diseases in cells or tissues.

Sphingosine kinase exists in a variety of cells with two types of isoforms, SphK1 and SphK2, and catalyzes the formation of S1P (Alemany et al., 2007) studies show that S1P is synthesized entirely by SphK *in vivo* and plays an important role in life activities (Mizugishi et al., 2005; Spiegel and Milstien, 2007). At present, the role of SphK1 in tissue fibrosis is clearer, while the studies on SphK2 are relatively few. SphK1 and SphK2 are located in different subcellular structures. SphK1 is mainly located in the cytoplasm and can be activated by a variety of agonists, including tumor necrosis factor (TNF)-α, vascular endothelial growth factor (VEGF), then transferred to the plasma membrane to convert the Sph to S1P (Taha et al., 2006). Conversely, SphK2 has the effect of inhibiting cell growth and promoting apoptosis (Igarashi et al., 2003).

Sphingosine 1-phosphate receptors are important participants in many life processes. S1P_1_ plays an important role in angiogenesis in the embryonic period (Liu et al., 2000), and its function in the regulation of blood pressure in adult individuals has also been confirmed (Ryu et al., 2002), and S1P_2_ is essential for the morphogenesis of zebrafish hearts (Kupperman et al., 2000). *In vivo*, the expression of S1PRs is regulated by many factors and cytokines. TGF-β1 has been proven to be an effective regulator to the expression of S1PRs (Chang et al., 2014). Using the supernatant of apoptotic cells to stimulate human macrophages can increase the expression level of S1P_1_ in macrophages (Weichand et al., 2013). A growing body of evidence support that S1PRs play an important role in profibrosis in a variety of cells (Schwalm et al., 2013). A study, using the model of S1PR2 null mice or S1PR3 and S1PR2 double null mice, finds that the activity of Rho which is closely related to fibrosis, decreases significantly in these mouse embryonic fibroblasts (Ishii et al., 2002). After inhibiting the expression of S1PR2 and S1PR3 genes *in vitro*, the differentiation of myofibroblasts is inhibited, and the study also localizes S1PR2 and S1PR3 on the cell membranes (Kono et al., 2007).

In order to combine with and activate S1PRs, a large amount of S1P needs to be secreted out of the cells by the transporters. This “inside-out-signaling” process involves the synthesis and transport of S1P in the cells and the activation of different kinds of S1PRs in the autocrine and paracrine signals in mammalian cells (Takabe and Spiegel, 2014). At present, confirmed S1P transporters are mainly a variety of ATP-binding cassette (ABC) transporters, which exist in a variety of cell lines (Mitra et al., 2006; Sato et al., 2007; Takabe et al., 2010). In addition, spinster homolog 2 (Spns2) may be a special S1P transporter in different tissues (Fukuhara et al., 2012; Nagahashi et al., 2013; Nishi et al., 2014).

Sphingosine 1-phosphate lyase is a key enzyme degrading S1P into the terminal molecule: hexadecenal and ethanolamine phosphate *in vivo* and *in vitro*, thereby controlling the concentrations of S1P [9]. Many experiments have demonstrated that activation of S1PL is involved in a variety of stimulation, such as bacterial endotoxin lipopolysaccharide (LPS) and radiation exposure (Zhao et al., 2011; Billich et al., 2013).

## The Relationship Between the S1P Signaling Pathway and Tissue Fibrosis

### Pulmonary Fibrosis

Pulmonary fibrosis is a lung disease characterized by alveolar epithelial damage and accumulation of myofibroblasts, which are caused by a variety of factors, such as infections, inflammation, radiation exposure, and environmental pollutants (Wynn and Ramalingam, 2012). In recent years, treatment of mice with bleomycin has been used to establish a model of lung fibrosis in mice. In addition, S1PR agonists, such as fingolimod (FTY720), are also often used in the construction of *in vitro* models of pulmonary fibrosis, because they can induce differentiation of lung fibroblasts to myofibroblasts under prolonged stimulation (Sobel et al., 2013). We would introduce further the function of FTY720 in studies on fibrosis diseases in later part of this article.

Idiopathic pulmonary fibrosis is a chronic and progressive lung disease, characterized by alveolar epithelial cell injury and areas of type II cell hyperplasia that results in the deposition of the ECM and the differentiation of fibroblasts to myofibroblasts (Selman et al., 2001). At present, there is no effective treatment for IPF except lung transplantation, and the average of survival only 3–5 years after diagnosis (Niederman and Peters, 1998). Although the molecular and cellular mechanisms of IPF are not completely understood, several factors have been identified to be involved in the progression of IPF, such as reactive oxygen species (ROS) (Wolters et al., 2014). S1P and the signaling pathway have been recently shown to promote the pathogenesis underlying IPF. A study showed that the S1P in bronchoalveolar lavage fluid (BALF) and serum of IPF patients increased significantly compared with the control group by microarray analysis of human peripheral blood mononuclear cells. Similarly, the increased expression of S1P, SphK1, and SphK2 was negatively correlated with lung function and survival rate (Milara et al., 2012). Also, it was demonstrated that the main pathological feature of IPF patients is cell proliferation, but not apoptosis and autophagy, which may be due to the high level of S1P inhibiting the pro-apoptosis effect caused by Cer (Scarpa et al., 2013). Therefore, inhibition of the formation of S1P may be an effective treatment for reducing the proliferation of fibroblasts in IPF patients.

In addition, results provided direct evidence that SphK1 may become a new type of target for treating pulmonary fibrosis. TGF-β is an important factor during the progress of pulmonary fibrosis, and S1P signaling pathways may be involved in the process of pulmonary fibrosis promotion by TGF-β. A previous study showed that the level of S1P regulated by TGF-β is also affected by the expression of SphK1 in lung fibrosis (Kono et al., 2007). SphK1 knockout or inhibiting SphK activity can reduce the expression of S1P and the secretion of TGF-β in cells, which can also reduce bleomycin-induced mouse pulmonary fibrosis (Huang et al., 2013a). Conversely, the preconditioning of anti-TGF-β antibody can inhibit the expression of S1PL mRNA and protein induced by TGF-β in human lung fibroblasts, which indicates that, in pulmonary fibrosis, the role of S1P and its signaling pathways also depends on the level of TGF-β (Huang and Natarajan, 2015). In the TGF-β1 induced WI-38 cell fibrosis model, TGF-β1 activates SphK1 and leads to the Rho-associated myofibroblast differentiation in fibrosis (Kono et al., 2007). SphK1-deficient mice were protected against bleomycin-induced pulmonary fibrosis, and the expression of SphK1 protein increased in the lung tissues of IPF patients compared with the control group (Huang et al., 2013b).

Sphingosine 1-phosphate receptors are also closely related to the development of lung fibrosis. WI-38 cells can express S1P_1_, S1P_2_, and S1P_3_ receptors *in vitro* (Urata et al., 2005). S1P can regulate pulmonary vascular permeability through S1P_1_, exogenous S1P, or S1P_1_ agonist activates S1P_1_ to mediate cytoskeletal rearrangements, promote intercellular adhesion and tight junction formation, enhance endothelial barrier function, and promote the development of fibrosis in endothelial cells. *In vitro*, the S1PR agonists such as S1P, FTY720-P, and TGF-β1 do not induce the expression of the myofibroblast marker α-SMA, but all can effectively stimulate the ECM synthesis and increase the expression of CTGF and other fibrosis markers. These results indicated that, different from TGF-β1, S1PR agonists activate PI3K/Akt and ERK1/2 signals, but not Smad2/3 signals, to mediate ECM synthesis, that is, S1PR agonists stimulate human pulmonary fibrosis by stimulating S1P_1_ and S1P_2_ in a Smad independent pathway (Sobel et al., 2013). Further studies have shown that short-term stimulation (24 h) of S1P_1_ agonists, such as FTY720 and AUY954, can enhance the integrity of the vascular endothelial barrier, reduce vascular leakage caused by injury, and protect the body. However, prolonging exposure time in these S1P_1_ agonists (>7 days) can aggravate mice pulmonary fibrosis and vascular permeability, which is induced by bleomycin (Sanchez et al., 2003; Shea et al., 2010). This effect requires further confirmation in *in vivo* experiments.

Sphingosine 1-phosphate lyase is also involved in fibrosis development. It was demonstrated that the expression of S1PL reduces TGF-induced differentiation of fibroblasts and inhibits the expression of S1PL by knocking out the gene attenuating pulmonary fibrosis, which is induced by bleomycin in mice. On the contrary, TGF-β increases the expression of S1PL in lung fibroblasts, and this effect can be inhibited by treating cells with anti-TGF-β antibody (Huang et al., 2015). Thus, S1PL may become a novel molecule to treatment lung fibrosis.

Autophagy has also been confirmed to be related to the process of lung fibrosis. Huang et al. (2015) structured a mouse model of partial deletion of S1PL gene and found that those mice reacted more violently to bleomycin-induced pulmonary fibrosis and the expression of autophagy-related markers, LC3 and beclin 1, are reduced when their lung S1P increases; however, the overexpressed S1PL can reduce S1P and increase the level of autophagy-related markers.

In general, S1P, SphK, S1P_1,2_, and S1PL have been proven to be involved in the development of pulmonary fibrosis, and the role it plays is clearer. Reducing the formation of S1P and inhibiting the activity of SphK and S1PL can effectively weaken the formation or development of fibrosis in both *in vivo* and *in vivo*, while the effects of using S1P_1_ and S1P_2_ agonists are related to the treatment time. At present, most of the pulmonary fibrosis diseases lack an effective treatment, and the role of S1P and its signaling pathway in pulmonary fibrosis is likely to be a potential therapeutic strategy.

### Liver Fibrosis

Liver fibrosis is a chronic progressive liver disease characterized by the accumulation of ECM protein (Bissell, 1998). It is a precursor of cirrhosis and even liver cancer caused by chronic liver disease, multiple injuries, or dysfunctional wound healing (Kisseleva et al., 2012). The development and remission of hepatic fibrosis is believed to be a reversible process (Kisseleva et al., 2012). At present, liver transplantation is the only treatment for terminal liver disease, but it is limited by the availability of donor organs and the risk of lifetime immunosuppression (Lucey et al., 2013). Bile duct ligation (BDL) and inducing chronic low-level inflammation and necrosis of the liver by CCl4 are often used in establishing hepatic fibrosis models, and the latter is considered to be closer to the morphological and pathophysiological state of human liver fibrosis (Marques et al., 2012).

Although the research on the etiology and molecular mechanism of liver fibrosis has made great progress in the past 20 years, there is still no effective treatment strategy. There is a comprehensive source of myofibroblasts in the liver, including hepatic stellate cells (HSCs) and bone marrow-derived mesenchymal stem cells (BMSCs) (Russo et al., 2006). At the early stages of the liver fibrosis, liver injury leads to the recruitment of inflammatory cells, which release many factors causing activation of HSCs. Activated HSCs turn into a myofibroblast-like phenotype, which gain contractile, proinflammatory, and fibrogenic properties and then release proteins leading to the accumulation of ECM (Flier and Underhill, 1993; Zhang et al., 2016). PDGF is the most effective mitogen and chemotactic factor of the HSCs released factors (Pinzani, 2002). Therefore, finding a way to block HSC activation may become an effective way to search for antifibrotic drugs. Stimulating liver regeneration may be another treatment strategy for liver diseases, and the use of HSCs is considered to be one of the potential therapeutic methods. In the advanced period of liver fibrosis, liver function is destroyed and irreversible cirrhosis develops (Rippe and Brenner, 2004).

In recent years, since S1P has been found to regulate the expression level of a variety of ECM in the process of liver fibrosis, it is believed to play an important role in the process of liver fibrosis by affecting the expression of various fibrotic markers (Li et al., 2009a). HSCs have a steady state of circulation in the body, and the gradient distribution of S1P in the tissues and lymph is identified as a key regulator of the cycle (Massberg et al., 2007). An *in vitro* study of CCl4-induced hepatic fibrosis shows that S1P promotes the development of liver fibrosis by participating in HSCs activation and differentiation to myofibroblasts (Gonzandez-Fernandez et al., 2017). At the same time, reducing the S1P liver tissue:lymph gradient by inhibiting the migration of HSCs can also preserve the liver and reduce subsequent fibrosis, which may be a new strategy in the treatment of hepatic fibrosis in future (King et al., 2017). An animal experiment shows that BMSCs in mice migrate to the damaged liver and activate myofibroblasts induced by S1P and its receptors after the liver is damaged (Yang et al., 2012). The movement of BMSCs has also been found to be regulated by the S1P concentration gradient in the liver and bone marrow (Li et al., 2009b). Hepatic fibrosis is a sign of performance during the pathogenesis of liver cancer. Recently, a cohort study showed that S1P may be one of markers to estimate the severity of liver disease (Bartels et al., 2017).

S1P_1_ and S1P_3_ are considered to be the two major S1PRs that play a key role in liver fibrosis. Exogenous S1P increases the expression of SphK1 and S1P_1_ and S1P_3_ in human HSCs LX-2 cells (Liu et al., 2011). This phenomenon also occurs in human fibrosis liver samples (Li et al., 2011). It has been shown that a potent agonist for four of the five S1PRs (S1P_1,3-5_), FTY720-P, plays a role in suppressing cell proliferation and migration in cultured HSCs by inhibiting the signal pathway between S1P and PDGF (Brunati et al., 2008). A study found that S1P-induced myofibroblasts migration from plasma to the liver injury depends on the role of S1P_3_ (Li et al., 2009a). A study showed that the level of S1P in the liver is closely related to the expression of angiogenesis markers Ang1 (angiopoietin) mRNA. In addition, the S1P_1_ and S1P_3_ antagonists block the upregulation of Ang1 and alleviate the degree of liver fibrosis in the damaged liver (Yang et al., 2013). HuR (human embryonic lethal abnormal visual protein) is a key post-transcriptional regulator of gene expression. In recent years, increasing attention has been paid to the role of HuR in fibrosis (Bai et al., 2012; Woodhoo et al., 2012). A study has shown that there is a positive feedback mechanism between HuR and S1P_3_, which can affect the vitality of BMSCs and further affect the process of fibrosis (Chang et al., 2017). However, although the expression of S1P and S1P_1_ and S1P_3_ is reported to play a role in promoting fibrosis in liver fibrosis models of BDL and CCl4 stimulation (Yang et al., 2013; Wang et al., 2015), S1P_1_ was associated with the formation of angiogenesis and granulation tissue in another report (Yanagida and Hla, 2016), and in a study of chronic injury and cholestasis in mice, S1P_1_ was found to be highly expressed in vascular endothelial cells and then reduce fibrosis by stimulating hepatic regeneration (Ding et al., 2016). These different results may be caused by different models of selection and may also be due to the removal of a variety of different kinds of liver cells, which lead to different stress responses. Therefore, in future studies, more specific experiments are needed to confirm the different roles of S1P and S1PRs in the process of fibrosis through different cells.

Studies about S1PRs and the differentiation of BMSCs are also contradictions. S1P_1_ and S1P_3_ are shown to be involved in the differentiation of BMSCs to myofibroblasts in mice (Yang et al., 2012). While they seem to inhibit the expression of collagen in human BMSCs (Chang et al., 2014). The above results from different laboratories may differ depending on the different species or models, but the direct mechanism of S1PRs in liver fibrosis requires further experimentation to verify, and its application in the treatment of liver fibrosis remains to be discussed.

The role of S1P_2_ in the process of fibrosis is also worthy of attention. The expression of S1P_2_ in the fibrosis case group and the control group is not significant in many experiments (Li et al., 2009b). However, in another study of the same model, the S1P_2_ gene knockout mice or those injected with S1P_2_ antagonist JTE-013 expressed low Rho kinase activity after BDL, and the expression of S1P_2_ mRNA increases after the operation in the wild-type mice (Kageyama et al., 2012). In addition, a recent study confirmed the role of S1P_2_ in the liver injury in a cholestasis mouse model (Wang et al., 2017). The hepatic fibrosis induced by CCl4 or dimethyl nitrosamine decreases in the S1P_2_^-/-^ mice (Ikeda et al., 2009), and the result of a population study shows that the expression of S1P_2_ decreases in patients with liver fibrosis (Li et al., 2011). These results suggest that S1P may play a role in fibrosis through S1P_2_ in the process of fibrosis.

Previous studies have shown that the S1P/SphK signaling pathway is involved in liver fibrosis depending on TGF-β. A recent study provided evidence that production of collagen I is increased by SphK1 in human liver myofibroblasts after TGF treatment (Xiu et al., 2015). Recently, Melatonin it was found to be involved in protecting fibrosis in different organs, including the liver (Fernández et al., 2015). A study used CCL4 to establish a liver fibrosis model and found that melatonin treatment not only reduces the degree of fibrosis, but also inhibits the SphK1/S1P signaling pathway both *in vitro* and *in vivo*, thus confirming the hypothesis (Gonzandez-Fernandez et al., 2017).

In addition, the effect of intracellular S1P in liver fibrosis is closely related to the role of transporter Spns2 (Xiu et al., 2015). S1P may be transported out of cells by Spns2 in the process of liver fibrosis, and then combines with S1PR to promote the liver fibrosis (Sato et al., 2016). All kinds of apolipoprotein play a similar role in bile and peripheral blood, and apoM is a kind of protein mainly derived from hepatocytes, in which the spatial structure determines that its main function is to carry lipid signal molecule S1P to bind with S1P_1-5_ on the membrane (Christoffersen et al., 2011; Książek et al., 2015; Hajny and Christoffersen, 2017). Research shows that apoM is the main carrier of S1P in plasma, and the secretion of apoM in the liver and other organs is correlated with the amount of S1P in the blood (Murata et al., 2000). A recent animal experiment has suggested that apoM is associated with S1P signaling pathways in liver fibrosis (Ding et al., 2016). Both S1P and apoM are involved in inducing the expression of liver receptor homologous 1 (LRH-1), suggesting that there might be a bile acid metabolic pathway between the two molecules (Pan et al., 2015). However, whether apoM is directly associated with S1P in plasma and secretion into bile needs to be explored further in the future.

In summary, the liver, as the most important metabolic organ in the body, has extensive blood flow and a complex metabolic system. Different from the lung, the concentration gradient of S1P in circulation and tissue seems to be more closely associated with liver fibrosis than the concentration of S1P, which may be due to the origin of macrophages, HSCs and BMSCs in the liver. The effect of the associated transporters in the S1P signaling pathway also contributes to the promotion of liver fibrosis. However, the role of S1PRs in liver fibrosis remains controversial and requires further study. In addition, the expression of TRAF2, melatonin, and other molecules are also involved in the regulation of liver fibrosis by S1P and its signaling pathway.

### Cardiac Fibrosis

Heart failure is one of the most important health problems worldwide. According to statistics, there are approximately 23 million heart failure patients all over the world (Amp and Wilkins, 2017), and the incidence and mortality of heart failure have been gradually increasing for several decades among people aged more than 65 years (Corrao and Maggioni, 2014). Myocardial fibrosis is one of the main manifestations of the development of heart failure. It is mainly due to the secondary diseases caused by ischemia-reperfusion injury or other causes. Fibroblasts can change from static state to proliferation, migration, and secretory state in the condition of myocardial infarction, which changes the phenotype to become myofibroblasts, and then expresses contractile proteins, including α-SMA, finally leading to myocardial fibrosis (Krenning et al., 2010). At present, there is no effective treatment for myocardial fibrosis (Opie et al., 2006). Therefore, studying its development process and treatment is the key to finding a treatment for heart failure.

In the cardiovascular system, the SM signaling pathway is considered to be a signal-regulated pathway in pathological conditions. A recent review article on the role of S1P in myocardium impairment suggested that a series of signaling pathways induced by S1P is the protective factor of myocardial ischemia reperfusion and oxidative injury, and HDL plays a role in promoting it (Karliner, 2009). However, the relationship between the protective effect and myocardial fibrosis is not mentioned in the article. Interestingly, both *in vitro* and *in vivo* experiments suggest that S1P is not involved in the process of mediating the hypertrophy of myocardial cells in neonatal mice (Sekiguchi et al., 1999; Takuwa et al., 2010). However, Philippe’s study suggests that S1P mediates myocardial cell hypertrophy in mice through S1P_1_ (Robert et al., 2001). A study has shown that S1P plays an important role in the regulation of cardiac fibroblast migration and proinflammatory response in myocardial hypoxia (Kacimi et al., 2007). Relaxin (RLX), a kind of peptide hormone causing a physiological cardiac effect, is the key regulation factor of ECM remodeling in many tissues (Et et al., 2008). A study using immature primary cardiac myocytes isolated from neonatal mice and mice heart myofibroblasts H9C2 found that RLX could mediate SM metabolism, SphK1 activation, and the production of S1P in cardiac myocytes. Meanwhile, the production of S1P mediated by RLX is the key to ECM remodeling in cardiac myocytes, and it provides a new therapeutic strategy for preventing cardiac fibrosis and promoting myocardial regeneration (Frati et al., 2015). However, studies researching the role of S1P in cardiac hypertrophy and fibrosis remain insufficient in both *in vitro* and *in vivo*, and the models are not consistent, and the model, which uses S1P or the interrelated molecules to induce myocardial hypertrophy and fibrosis directly, is likely to change the metabolic rate of other molecules in the pathway, resulting in incorrect judgment of the effects of S1P. Thereby, these conclusions suggest that using S1P and some molecules in its pathway may be a potential method for the treatment of myocardial hypertrophy and fibrosis, while, due to the contrary results, the mechanism still needs to be further confirmed in *in vivo* experiments, and the correlation between these phenomena and human heart fibrosis warrants further exploration.

The expression of SphK1 in cardiac fibroblasts can affect the degeneration and fibrosis of cardiomyocytes. *In vivo* study showed that SPHK1-transgenic mice present myocardial degeneration and fibrosis at high levels of SphK1 (SphK1 activity increased 20 times), while this is not observed at lower SphK1 levels (SphK1 activity increased five times) (Takuwa et al., 2010). However, there is an opposite conclusion: the expression of SphK1 in cardiac fibroblasts is much higher than that in cardiac myocytes. Under normal conditions, SphK1 is necessary for the proliferation of cardiac fibroblasts, but in the case of myocardial hypoxia, SphK1 plays an anti-inflammatory role and inhibits the occurrence of cardiac fibrosis. Therefore, it plays a dual regulatory role in cardiac physiology and pathology (Kacimi et al., 2007). The above two studies have shown that SphK1 does play a regulatory role in the process of cardiac fibrosis. However, this effect may be related to the activity and expression of SphK1. Therefore, the mechanisms of SphK1 in cardiac fibrosis require further study.

S1P_1_, S1P_2_, and S1P_3_ are expressed in the heart. There are differences in the distribution of different subtypes of S1PRs in different types of cardiac cells: the main distribution of S1PR in cardiomyocytes is S1P_1_, while the expression of S1P_2_ and S1P_3_ are low (Zhang et al., 2007; Landeen et al., 2008), and S1P_3_ is mainly distributed in fibroblasts (Landeen et al., 2007). A study shows that S1PRs can regulate the ion channel and mediate myocardial self-protection through ischemic preconditioning, so it is a key molecule to regulate the function of the myocardium (Keul et al., 2016). A mouse experiment shows that the expression of S1P_1_ is upregulated during myocardial hypertrophy and could be overexpressed in interleukin (IL)-6 secretion depending on the Ang II-AT1, causing cardiac hypertrophy and fibrosis, and affecting the systolic function of the heart. However, this phenomenon occurs only in the proliferative fibroblasts/myocytes of the mouse heart, but not in the cardiomyocytes (Ohkura et al., 2017). *In vivo*, the activation of S1P_2_ and S1P_3_ can weaken the ischemia-reperfusion injury of myocardium. The mechanism may be that S1P can affect ventricular fibrosis through S1P_3_, and then mediate fibroblast differentiation into myofibroblasts, which may be a therapeutic strategy to treat fibrosis after cardiac physiology and pathology injury (Means et al., 2007; Benamer et al., 2011). Additionally, knocking out S1P_3_ in the heart cells can inhibit the fibrosis in mice with high expression of SphK1 through a Rho- and Smad3-dependent signaling pathway (Takuwa et al., 2010). FTY720 can also reduce the formation of myocardial fibrosis by inhibiting oxygen free radicals to produce strong anti-inflammatory and antioxidant properties, thus reducing the death of cardiomyocytes (Aytan et al., 2016). A study using the heart transplantation mouse model suggests that FTY720 is an effective ischemic preconditioning agent, which can reduce apoptosis, inflammation, oxidative stress, and myocardial fibrosis in the model (Ahmed et al., 2017).

In recent years, the role of S1P in the relationship between autophagy and myocardial damage has become a topic of concern. As in other tissues, autophagy after myocardial damage is beneficial to the organism in most cases, but in some cases, it also causes damage to the organism. A study suggests that autophagy-related survival is regulated by SphK1 to some extent, while autophagy-related deaths are regulated by Cer (Lavieu et al., 2007).

In a comprehensive sense, S1P, S1PR, and SphK are related to the expression of several factors in many interrelated fibrotic pathways in the process of cardiac fibrosis and play a role in the pathogenesis and development of fibrosis together. However, the molecular mechanism of the relationship between S1P and cardiac fibrosis is unclear, as is the promotion or inhibition of cardiac fibrosis in different conditions. Therefore, whether these molecules in the pathway can be used in future as a target for the treatment of cardiac fibrosis still require further study *in vitro* and *in vivo*.

### Other Tissue Fibrosis

Diabetic nephropathy, which usually leads to renal fibrosis, begins with hyperglycemia, which subsequently generates glucotoxicity in renal tubular epithelial cells. Later, like other fibrosis diseases, inflammatory cells are recruited and activated to produce a large number of inflammatory factors, such as monocyte chemoattractant protein 1 (MCP1) and TGF-βs, which are soon released into the interstitium, and fibrosis-related signal pathways are activated, resulting in the positive feedback to promote the production and accumulation of ECM. In particular, extracellular growth factors can stimulate the paracrine effect in renal fibrosis (Lehmann and Schleicher, 2000). A study has shown that S1P signaling may take part in the development of fibrosis in diabetic nephropathy (Yaghobian et al., 2016). Living in the environment of hyperglycemia and oxidative stress for a long time activate SphK1 and increase S1P synthesis (Wang et al., 2005). Glucose stimulation increased the level of S1P in pancreatic islet cells under the action of SphK2, but the levels of Cer and Sph remained unchanged (Boslem et al., 2012; Stanford et al., 2012). A further study has shown that FTY720 selectively inhibits lymphocyte migration and alleviates ischemia-reperfusion injury after renal fibrosis by reducing the release of ECM and finally reduces the progression of glomerulosclerosis (Delbridge et al., 2007).

Gluteal muscle contracture (GMC) is also a chronic fibrotic disease caused by multiple etiologies, which is characterized by proliferation of fibroblasts and accumulation of collagen in the ECM of muscles (Zhao et al., 2010). A recent study provided evidence that the level of S1P increased in the muscles of patients with GMC, and exogenous S1P stimulation can promote the proliferation of contraction band (CB) fibroblasts in a dose- and time-dependent manner, and also promote the expression of α-SMA, collagen, TGF-β1, and PAI-1 at mRNA and protein levels, which indicates that S1P plays several roles in the development of GMC (Zhao et al., 2013). In addition, the SphK1/S1PR signaling pathway is also involved in the process of TGF-β-mediated skeletal muscle fibroblasts transforming into myofibroblasts and plays a role in profibrosis (Cencetti et al., 2010).

Sphingosine 1-phosphate and the related signaling pathway are also involved in ocular fibrosis. It can stimulate retinal pigmented epithelial cells converting into fibroblast-like cells. At the same time, S1P increases two markers of fibrosis, PAI-1 and HSP47 (Swaney et al., 2008). Intravenous anti-S1P monoclonal antibody can inhibit choroid neovascularization and collagen deposition under the retina, suggesting that extracellular S1P plays an important role in ocular fibrosis (Caballero et al., 2009). Therefore, the use of anti-S1P antibodies may be a therapeutic strategy for ocular fibrosis and exudative age-related macular degeneration by inhibiting the formation of profibrosis factors, angiogenesis, and retinal fibrosis, but research is still lacking regarding the role of S1PRs.

Dermal fibroblasts and myofibroblasts play an important role in the repair and remodeling of wounds. Due to the role of S1P and S1PRs in cell migration and their profibrosis effects, a study has suggested that they may have important clinical implications in trauma repair and abnormal wound healing (Watterson et al., 2007). S1P_1_ and S1P_3_ have been shown to cooperate with TLR4 to promote the secretion of pro-inflammatory cytokines in endothelial and epithelial cells (Eskan et al., 2008). An *in vitro* experiment demonstrated that TLR activation and S1P stimulation together regulate the inflammatory response in normal human dermal fibroblasts, which has been shown to induce pro-inflammatory cytokine production and myofibroblast differentiation as well as cell migration (Hamidi et al., 2014).

In general, S1P is up-regulated in the development of kidney, gluteal, ocular, and dermal fibrosis, and is thought to play a role in promoting fibrosis by interacting with other molecules, such as inflammatory factors, in these tissue fibrosis.

## Clinical Application of S1P Signaling

As mentioned earlier, extensive studies point out that the role of S1P in fibrosis comes from if it actives S1PRs through the actions of S1P in the context of autocrine or paracrine signaling or directly participates in the signal transduction in the cell.

Sphingosine 1-phosphate, which is secreted to the ECM, promotes fibrosis mainly through S1P_2_- and S1P_3_-mediated TGF-β and other signaling pathways in many organs or *in vitro* cell lines. Therefore, antagonizing S1P_2_ or S1P_3_ may be measurable interventions to prevent fibrosis. However, only little S1PRs antagonists have been used in clinic settings.

FTY720 was originally used as a kind of immunosuppressant, and it can induce a significant reduction of peripheral lymphocytes by inducing the lymphocytic closure in secondary lymphoid organs. In recent years, its role in fibrosis has gradually been taken more seriously. *In vivo*, FTY720 can be rapidly phosphorylated into FTY720-P, which shares structural homology to the natural ligand S1P, so that is highly affinity with S1P_1_ and S1P_3-5_ and can be combined with them (Brinkmann et al., 2002). Therefore, FTY720 is widely used in fibrosis-related studies. For example, in the studies of fibrotic animal models, FTY720 plays a useful role in the treatment of fibrosis by reducing the inflammatory response or inhibiting the dysfunction of the microvascular endothelium (Liu et al., 2013). Clinically, it is used for oral treatment of relapsing multiple sclerosis because it can promote cell phagocytosis and the degradation of S1P, thereby antagonizing the role of S1P in cells (Brinkmann et al., 2010). However, FTY720 is also used in the establishment of fibrotic models in both *in vitro* and animal experiments. Currently, it is believed that the phosphorylated form FTY720-P causes desensitization of the receptor, that is, cells or tissues exposed to FTY720-P for a long time are inactivated to S1P, showing a phenotype similar to the lack of circulating S1P or the treatment of S1PRs antagonists. Therefore, the clinical use of FTY720 should be very cautious and fully considered about its risk of prolonged treatment.

In the lung and cardiac fibroblasts, SphK1 mediates TGF-β-activated profibrosis to produce S1P. Then, those S1Ps are released out of cells to bind with S1P_2_ or S1P_3_, and finally promote fibroblast differentiation (Kono et al., 2007; Lowe et al., 2009). In the meantime, inhibition of SphK1 may also play a role in inhibiting fibrosis by inducing fibroblast apoptosis (Pyne and Pyne, 2017). Therefore, the double-inhibition of SphK1 and the antagonistic effect of S1P_2_ and S1P_3_ may be effective means of reducing fibrosis.

However, some studies thinks that intracellular S1P is an anti-fibrotic molecule. Whether this effect is directly caused by S1P and then induces the change of Cer, or by the change of ratio between the two molecules remains to be further verified. Therefore, due to the potential protective role of S1P in cells, the molecular mechanism of SphK1 inhibitors to prevent fibrosis will become more complex.

In recent years, the role of dhS1P in fibrosis has been gradually concerned. Similar to S1P, dhS1P is catalyzed by SphK, while the expression of dhS1P is much lower than S1P *in vivo* and the function is not completely clear. DhS1P and S1P play different roles in the fibrosis by participating in different signaling pathways. The increased expression of S1P can promote the expression of tissue inhibitor of metalloproteinase-1 (TIMP-1) induced by TGF-β and then inhibit the degradation of ECM and promote the occurrence of fibrosis (Yamanaka et al., 2004). DhS1P can stimulate the generation of matrix metalloproteinase 1 (MMP1), which induces the degradation of ECM and has the opposite biological function to S1P (Long et al., 2010). The results from the study of fibroblast in patients with SSC also confirmed that dhS1P may be a potential drug for the treatment of fibrosis (Bu et al., 2010). However, both S1P and dhS1P are catalyzed by SphK, and all of them play a biological role by activating S1PRs. Therefore, further experiments are needed to determine how they play different roles in the process of fibrosis and whether they combine with the receptors, leading to different conformational changes of the receptor to activate different signals. It is possible to find a new intervention target for the treatment of fibrosis in this direction.

In addition, the different roles of inflammatory cells and those secreted cytokines in different stages of fibrosis are also noteworthy. For example, macrophages associated with inflammation and fibrosis plays an opposite role in lung inflammation (the early stages of fibrosis) and tissue remodeling (the late stages of fibrosis) (Li et al., 2009c). Several inflammatory factors, such as TNF-α, promote fibrosis through stimulating the synthesis of IL-1β and TGF-β1 in a variety of cells, including macrophages and fibroblasts (Tsoutsou and Koukourakis, 2006; Wynn, 2011) but inhibit collagen synthesis in myofibroblasts (Siwik et al., 2000). However, there are relatively few studies on the different roles of S1P and the related signaling pathways in different stages of fibrosis. Therefore, it is necessary for us to better understand the role of each kind of cell, molecule, and element in the different stages of fibrosis and thus to improve the molecular mechanism of the inflammatory and proliferative responses to fibrosis, then better utilize the S1P and its signaling pathways in the treatment of fibrosis-related diseases.

In addition to drug therapy, S1P is also a potential biomarker of some diseases. In a study, S1P was considered to be a potential biomarker for judging the severity of liver diseases (Bartels et al., 2017). However, due to the complexity and high cost during the measure of S1P at present, the clinical application cannot be realized in a short time. Therefore, it is necessary to explore an efficient, simple, and economical method of detecting S1P.

## Conclusion

At present, an effective treatment of fibrosis is still lacking. Once the fibrous tissue has formed, it is difficult to repair into normal tissue. Therefore, the studies on preventing or slowing down the development of fibrosis diseases and blocking the upstream biological processes before fibrogenesis offer better strategies for these kinds of diseases at present. A large number of *in vitro* and *in vivo* studies have shown that S1P and its signaling pathways are involved in the development of multiple tissue fibrosis. Through combining with different S1PRs, S1P may play different roles in different organs and models. SphK, S1PL, and several kinds of S1P transporters also take part in the process of fibrosis by regulating the synthesis, degradation, and transfer of S1P. At the same time, S1P and the related signaling pathways compose complex signaling networks with so many signal pathways, such as inflammation, apoptosis, and autophagy, and regulate the development of fibrosis. However, the mechanism of S1P and the related signaling pathways in fibrosis have not been fully elucidated, and they have different roles in different organs and species that require further study. In addition, the intervention of S1P and the related signaling pathways are potential treatments for fibrosis-related diseases, and S1P is also expected to become an effective biomarker of the severity of fibrosis diseases in the future.

## Author Contributions

EW contributed to the preparation and writing of the manuscript. MZ and XH reviewed and edited the contents of the manuscript.

## Conflict of Interest Statement

The authors declare that the research was conducted in the absence of any commercial or financial relationships that could be construed as a potential conflict of interest.

## References

[B1] Ahmed N. Linardi D. Muhammad N. Chiamulera C. Fumagalli G. Biagio L. S. (2017). Sphingosine 1-phosphate receptor modulator fingolimod (FTY720) attenuates myocardial fibrosis in post-heterotopic heart transplantation. *Front. Pharmacol.* 8:645. 10.3389/fphar.2017.00645 28966593 PMC5605636

[B2] Alemany R. van Koppen C. J. Danneberg K. Ter B. M. Meyer Z. H. D. (2007). Regulation and functional roles of sphingosine kinases. *Naunyn Schmiedebergs Arch. Pharmacol.* 374 413–428. 17242884 10.1007/s00210-007-0132-3

[B3] Amp L. Wilkins A. (2017). Correction to: heart disease and stroke statistics-2017 update: a report from the american heart association. *Circulation* 135:e646. 10.1161/CIR.0000000000000491 28264899

[B4] Aytan N. Choi J. K. Carreras I. Brinkmann V. Kowall N. W. Jenkins B. G. (2016). Fingolimod modulates multiple neuroinflammatory markers in a mouse model of Alzheimer’s disease. *Sci. Rep.* 6:24939. 10.1038/srep24939 27117087 PMC4847010

[B5] Bai D. Gao Q. Li C. Lan G. Gao Y. Wang H. (2012). A conserved TGFβ1/HuR feedback circuit regulates the fibrogenic response in fibroblasts. *Cell. Signal.* 24 1426–1432. 10.1016/j.cellsig.2012.03.003 22446588

[B6] Bartels M. Scholz M. Becker S. Kinny-ko B. Seehofer D. Berg T. (2017). Low sphingosine-1-phosphate plasma levels are predictive for increased mortality in patients with liver cirrhosis. *PLoS One* 12:e0174424. 10.1371/journal.pone.0174424 28334008 PMC5363961

[B7] Benamer N. Fares N. Bois P. Faivre J. (2011). Electrophysiological and functional effects of sphingosine-1-phosphate in mouse ventricular fibroblasts. *Biochem. Biophys. Res. Commun.* 408 6–11. 10.1016/j.bbrc.2011.03.072 21420933

[B8] Bhavanam N. P. Athena F. Young C. Lockey R. F. Narasaiah K. (2014). Commentary: the sphingosine kinase 1/sphingosine-1-phosphate pathway in pulmonary arterial hypertension. *Am. J. Respir. Crit. Care Med.* 190 1032–1043. 10.1164/rccm.201401-0121OC 25180446 PMC4299585

[B9] Billich A. Baumruker T. Beerli C. Bigaud M. Bruns C. Calzascia T. (2013). Partial deficiency of sphingosine-1-phosphate lyase confers protection in experimental autoimmune encephalomyelitis. *PLoS One* 8:e59630. 10.1371/journal.pone.0059630 23544080 PMC3609791

[B10] Bissell D. M. (1998). Hepatic fibrosis as wound repair: a progress report. *J. Gastroenterol.* 33 295–302. 10.1007/s005350050087 9605966

[B11] Blaho V. A. Galvani S. Engelbrecht E. Liu C. Swendeman S. L. Kono M. (2015). HDL-bound sphingosine 1-phosphate restrains lymphopoiesis and neuroinflammation. *Nature* 523 342–346. 10.1038/nature14462 26053123 PMC4506268

[B13] Boslem E. Meikle P. J. Biden T. J. (2012). Roles of ceramide and sphingolipids in pancreatic β-cell function and dysfunction. *Islets* 4 177–187. 10.4161/isl.20102 22847494 PMC3442815

[B14] Brinkmann V. Billich A. Baumruker T. Heining P. Schmouder R. Francis G. (2010). *Fingolimod (FTY720): Discovery and Development of an Oral Drug to Treat Multiple Sclerosis*. Hoboken, NJ: Blackwell Publishers. 10.1038/nrd3248 21031003

[B15] Brinkmann V. Davis M. D. Heise C. E. Albert R. Cottens S. Hof R. (2002). The immune modulator FTY720 targets sphingosine 1-phosphate receptors. *J. Biol. Chem.* 277 21453–21457. 10.1074/jbc.C200176200 11967257

[B16] Brunati A. M. Tibaldi E. Carraro A. Gringeri E. D’Amico F. Toninello A. (2008). Cross-talk between PDGF and S1P signalling elucidates the inhibitory effect and potential antifibrotic action of the immunomodulator FTY720 in activated HSC-cultures. *Biochim. Biophys. Acta Mol. Cell Res.* 1783 347–359. 10.1016/j.bbamcr.2007.11.008 18157950

[B17] Bu S. Asano Y. Bujor A. Highland K. Hant F. Trojanowska M. (2010). Dihydrosphingosine 1-phosphate has a potent antifibrotic effect in scleroderma fibroblasts via normalization of phosphatase and tensin homolog levels. *Arthritis Rheum.* 62 2117–2126. 10.1002/art.27463 20309867 PMC3034368

[B18] Caballero S. Swaney J. Moreno K. Afzal A. Kielczewski J. Stoller G. (2009). Anti-sphingosine-1-phosphate monoclonal antibodies inhibit angiogenesis and sub-retinal fibrosis in a murine model of laser-induced choroidal neovascularization. *Exp. Eye Res.* 88 367–377. 10.1016/j.exer.2008.07.012 18723015 PMC3756152

[B150] Cencetti F. Bernacchioni C. Nincheri P. Donati C. Bruni P. (2010). Transforming growth factor-beta1 induces transdifferentiation of myoblasts into myofibroblasts via up-regulation of sphingosine kinase-1/S1P3 axis. *Mol. Biol. Cell* 21, 1111–1124. 10.1091/mbc.E09-09-0812 20089836 PMC2836962

[B19] Chang N. Ge J. Xiu L. Zhao Z. Duan X. Tian L. (2017). HuR mediates motility of human bone marrow-derived mesenchymal stem cells triggered by sphingosine 1-phosphate in liver fibrosis. *J. Mol. Med.* 95 69–82. 10.1007/s00109-016-1460-x 27543493

[B20] Chang N. Xiu L. Li L. (2014). Sphingosine 1-phosphate receptors negatively regulate collagen type I/III expression in human bone marrow-derived mesenchymal stem cell. *J. Cell. Biochem.* 115 359–367. 10.1002/jcb.24670 24038457

[B21] Christoffersen C. Obinata H. Kumaraswamy S. B. Galvani S. Ahnström J. Sevvana M. (2011). Endothelium-protective sphingosine-1-phosphate provided by HDL-associated apolipoprotein M. *Proc. Natl. Acad. Sci. U.S.A.* 108 9613–9618. 10.1073/pnas.1103187108 21606363 PMC3111292

[B22] Corrao G. Maggioni A. P. (2014). Epidemiology of heart failure. *G. Ital. Cardiol.* 15(2 Suppl. 2) 10S–15S. 10.1714/1465.16183 24770484

[B23] Cyster J. G. Schwab S. R. (2012). Sphingosine-1-phosphate and lymphocyte egress from lymphoid organs. *Annu. Rev. Immunol.* 30 69–94. 10.1146/annurev-immunol-020711-075011 22149932

[B24] Delbridge M. Shrestha B. M. El-Nahas A. Haylor J. (2007). FTY720 reduces extracellular matrix expansion associated with ischemia-reperfusion induced injury. *Transplant. Proc.* 39 2992–2996. 10.1016/j.transproceed.2007.04.027 18089307

[B25] Delgado A. Fabrias G. Bedia C. Casas J. Abad J. L. (2012). Sphingolipid modulation: a strategy for cancer therapy. *Anticancer Agents Med. Chem.* 12 285–302. 10.2174/187152012800228643 21864240

[B26] Ding B.-S. Liu C. H. Sun Y. Chen Y. Swendeman S. L. Jung B. (2016). HDL activation of endothelial sphingosine-1-phosphate receptor-1 (S1P1) promotes regeneration and suppresses fibrosis in the liver. *JCI Insight* 1:e87058. 10.1172/jci.insight.87058 28018969 PMC5161208

[B27] Donoviel M. S. Hait N. C. Ramachandran S. Maceyka M. Takabe K. Milstien S. (2015). Spinster 2, a sphingosine-1-phosphate transporter, plays a critical role in inflammatory and autoimmune diseases. *FASEB J.* 29 5018-5028. 10.1096/fj.15-274936 26324848 PMC4653056

[B28] Eskan M. A. Rose B. G. Benakanakere M. R. Zeng Q. Fujioka D. Martin M. H. (2008). TLR4 and S1P receptors cooperate to enhance inflammatory cytokine production in human gingival epithelial cells. *Eur. J. Immunol.* 38 1138–1147. 10.1002/eji.200737898 18395849 PMC2738989

[B29] Et V. D. W. Halls M. L. Samuel C. S. Bathgate R. A. Unemori E. N. Sutton S. W. (2008). Relaxin family peptide receptors–from orphans to therapeutic targets. *Drug Discov. Today* 13 640–651. 10.1016/j.drudis.2008.04.002 18675759

[B30] Fernández A. Ordóñez R. Reiter R. J. Gonzálezgallego J. Mauriz J. L. (2015). Melatonin and endoplasmic reticulum stress: relation to autophagy and apoptosis. *J. Pineal Res.* 59 292–307. 10.1111/jpi.12264 26201382

[B31] Flier J. S. Underhill L. H. (1993). The cellular basis of hepatic fibrosis – mechanisms and treatment strategies. *N. Engl. J. Med.* 328 1828–1835. 10.1056/NEJM199306243282508 8502273

[B32] Frati A. Ricci B. Pierucci F. Nistri S. Bani D. Meacci E. (2015). Role of sphingosine kinase / S1P Axis in ECM remodeling of cardiac cells elicited by relaxin. *Mol. Endocrinol.* 29 53–67. 10.1210/me.2014-1201 25415609 PMC5414773

[B33] Fukuhara S. Simmons S. Kawamura S. Inoue A. Orba Y. Tokudome T. (2012). The sphingosine-1-phosphate transporter Spns2 expressed on endothelial cells regulates lymphocyte trafficking in mice. *J. Clin. Invest* 122 1416–1426. 10.1172/JCI60746 22406534 PMC3314466

[B34] Galvani S. Sanson M. Blaho V. A. Swendeman S. L. Obinata H. Conger H. (2015). HDL-bound sphingosine 1-phosphate acts as a biased agonist for the endothelial cell receptor S1P1 to limit vascular inflammation. *Sci. Signal.* 8:ra79. 10.1126/scisignal.aaa2581 26268607 PMC4768813

[B35] Gonzandez-Fernandez B. I Sanchez D. Crespo I. (2017). Inhibition of the SphK1/S1P signaling pathway by melatonin in mice with liver fibrosis and human hepatic stellate cells. *Int. Union Biochem. Mol. Biol.* 43 272–282. 10.1002/biof.1342 27801960

[B36] Hajny S. Christoffersen C. (2017). A novel perspective on the ApoM-S1P axis, highlighting the metabolism of apom and its role in liver fibrosis and neuroinflammation. *Int. J. Mol. Sci.* 18:e1636. 10.3390/ijms18081636 28749426 PMC5578026

[B37] Hamidi S. Schäfer-Korting M. Weindl G. (2014). TLR2/1 and sphingosine 1-phosphate modulate inflammation, myofibroblast differentiation and cell migration in fibroblasts. *Biochim. Biophys. Acta Mol. Cell Biol. Lipids* 1841 484–494. 10.1016/j.bbalip.2014.01.008 24440818

[B38] Hla T. Lee M. J. Ancellin N. Paik J. H. Kluk M. J. (2001). Lysophospholipids–receptor revelations. *Science* 294 1875–1878. 10.1126/science.1065323 11729304

[B39] Huang L. S. Berdyshev E. Mathew B. Fu P. Gorshkova I. A. He D. (2013a). Targeting sphingosine kinase 1 attenuates bleomycininduced pulmonary fibrosis. *FASEB J.* 27 1749–1760. 10.1096/fj.12-219634 23315259 PMC3606540

[B40] Huang L. S. Fu P. Patel P. Harijith A. Sun T. Zhao Y. (2013b). Lysophosphatidic acid receptor–2 deficiency confers protection against bleomycin-induced lung injury and fibrosis in mice. *Am. J. Respir. Cell Mol. Biol.* 49 912–922. 10.1165/rcmb.2013-0070OC 23808384 PMC3931116

[B41] Huang L. S. Berdyshev E. V. Tran J. T. Xie L. Chen J. Ebenezer D. L. (2015). Sphingosine-1-phosphate lyase is an endogenous suppressor of pulmonary fibrosis: role of S1P signalling and autophagy. *Thorax* 70 1138–1148. 10.1136/thoraxjnl-2014-206684 26286721

[B42] Huang L. S. Natarajan V. (2015). Sphingolipids in pulmonary fibrosis. *Adv. Biol. Regul.* 57 55–63. 10.1016/j.jbior.2014.09.008 25446881 PMC4291314

[B43] Igarashi N. Okada T. Hayashi S. Fujita T. Jahangeer S. Nakamura S. (2003). Sphingosine kinase 2 is a nuclear protein and inhibits DNA synthesis. *J. Biol. Chem.* 278 46832–46839. 10.1074/jbc.M306577200 12954646

[B44] Ikeda H. Watanabe N. Ishii I. Shimosawa T. Kume Y. Tomiya T. (2009). Sphingosine 1-phosphate regulates regeneration and fibrosis after liver injury via sphingosine 1-phosphate receptor 2. *J. Lipid Res.* 50 556–564. 10.1194/jlr.M800496-JLR200 18955732 PMC2638109

[B45] Ishii I. Ye X. Friedman B. Kawamura S. Contos J. J. Kingsbury M. A. (2002). Marked perinatal lethality and cellular signaling deficits in mice null for the two sphingosine 1-phosphate (S1P) receptors, S1P(2)/LP(B2)/EDG-5 and S1P(3)/LP(B3)/EDG-3. *J. Biol. Chem.* 277 25152–25159. 10.1074/jbc.M200137200 12006579

[B46] Kacimi R. Vessey D. A. Honbo N. Karliner J. S. (2007). Adult cardiac fibroblasts null for sphingosine kinase-1 exhibit growth dysregulation and an enhanced proinflammatory response. *J. Mol. Cell. Cardiol.* 43 85–91. 10.1016/j.yjmcc.2007.04.007 17512943

[B47] Kageyama Y. Ikeda H. Watanabe N. Nagamine M. Kusumoto Y. Yashiro M. (2012). Antagonism of sphingosine 1-phosphate receptor 2 causes a selective reduction of portal vein pressure in bile duct-ligated rodents. *Hepatology* 56 1427–1438. 10.1002/hep.25780 22505286

[B48] Karliner J. S. (2009). Sphingosine kinase and sphingosine 1-phosphate in cardioprotection. *J. Cardiovasc. Pharmacol.* 53 189–197. 10.1097/FJC.0b013e3181926706 19247197 PMC2835544

[B49] Keul P. Van Borren M. M. Ghanem A. Müller F. U. Baartscheer A. Verkerk A. O. (2016). Sphingosine-1-phosphate receptor 1 regulates cardiac function by modulating Ca2+ sensitivity and Na+/H+ exchange and mediates protection by ischemic preconditioning. *J. Am. Hear. Assoc.* 5:e003393. 10.1161/JAHA.116.003393 27207969 PMC4889204

[B50] King A. Houlihan D. D. Kavanagh D. Haldar D. Luu N. Owen A. (2017). Sphingosine-1-phosphate prevents egress of hematopoietic stem cells from liver to reduce fibrosis. *Gastroenterology* 153 233.e16–248.e16. 10.1053/j.gastro.2017.03.022 28363640 PMC5511862

[B51] Kisseleva T. Cong M. Paik Y. Scholten D. Jiang C. Benner C. (2012). Myofibroblasts revert to an inactive phenotype during regression of liver fibrosis. *Proc. Natl. Acad. Sci. U.S.A.* 109 9448–9453. 10.1073/pnas.1201840109 22566629 PMC3386114

[B52] Kong Y. Hong W. Wang S. Na T. (2014). FTY720, a sphingosine-1 phosphate receptor modulator, improves liver fibrosis in a mouse model by impairing the motility of bone marrow-derived mesenchymal stem cells. *Inflammation* 37 1326–1336. 10.1007/s10753-014-9877-2 24682874

[B53] Kono Y. Nishiuma T. Nishimura Y. Kotani Y. Okada T. Nakamura S. (2007). Sphingosine kinase 1 regulates differentiation of human and mouse lung fibroblasts mediated by TGF-beta1. *Am. J. Respir. Cell Mol. Biol.* 37 395–404. 10.1165/rcmb.2007-0065OC 17641298

[B54] Krenning G. Zeisberg E. M. Kalluri R. (2010). The origin of fibroblasts and mechanism of cardiac fibrosis. *J. Cell. Physiol.* 225 631–637. 10.1002/jcp.22322 20635395 PMC3098503

[B55] Książek M. Chacińska M. Chabowski A. Baranowski M. (2015). Sources, metabolism, and regulation of circulating sphingosine-1-phosphate. *J. Lipid Res.* 56 1271–1281. 10.1194/jlr.R059543 26014962 PMC4479332

[B56] Kupperman E. An S. Osborne N. Waldron S. Stainier D. Y. (2000). A sphingosine-1-phosphate receptor regulates cell migration during vertebrate heart development. *Nature* 406 192–195. 10.1038/35018092 10910360

[B57] Landeen L. K. Aroonsakool N. Haga J. H. Hu B. S. Giles W. R. (2007). Sphingosine-1-phosphate receptor expression in cardiac fibroblasts is modulated by *in vitro* culture conditions. *Am. J. Physiol. Hear. Circ. Physiol.* 292 H2698. 10.1152/ajpheart.01065.2006 17337593

[B58] Landeen L. K. Dederko D. A. Kondo C. S. Hu B. S. Aroonsakool N. Haga J. H. (2008). Mechanisms of the negative inotropic effects of sphingosine-1-phosphate on adult mouse ventricular myocytes. *Am. J. Physiol. Hear. Circ. Physiol.* 294 H736–H749. 10.1152/ajpheart.00316.2007 18024550

[B59] Lavieu G. Scarlatti F. Sala G. Levade T. Ghidoni R. Botti J. (2007). Is autophagy the key mechanism by which the sphingolipid rheostat controls the cell fate decision? *Autophagy* 3 45–47. 17035732 10.4161/auto.3416

[B60] Lehmann R. Schleicher E. D. (2000). Molecular mechanism of diabetic nephropathy. *Clin. Chim. Acta* 297 135–144. 10.1016/S0009-8981(00)00240-0 10841915

[B61] Ley B. Collard H. R. King Jr (2011). Clinical course and prediction of survival in idiopathic pulmonary fibrosis. *Am. J. Respir. Crit. Care Med.* 183 431–440. 10.1164/rccm.201006-0894CI 20935110

[B62] Li C. Jiang X. Yang L. Liu X. Yue S. Li L. (2009a). Involvement of sphingosine 1-phosphate (SIP)/S1P3 signaling in cholestasis-induced liver fibrosis. *Am. J. Pathol.* 175 1464–1472. 10.2353/ajpath.2009.090037 19729475 PMC2751543

[B63] Li C. Kong Y. Wang H. Wang S. Yu H. Liu X. (2009b). Homing of bone marrow mesenchymal stem cells mediated by sphingosine 1-phosphate contributes to liver fibrosis. *J. Hepatol.* 50 1174–1183. 10.1016/j.jhep.2009.01.028 19398237

[B64] Li M. Abdollahi A. Gröne H. Lipson K. E. Belka C. Huber P. E. (2009c). Late treatment with imatinib mesylate ameliorates radiation-induced lung fibrosis in a mouse model. *Radiat. Oncol.* 4:66. 10.1186/1748-717X-4-66 20025728 PMC2802357

[B65] Li C. Zheng S. You H. Liu X. Lin M. Yang L. (2011). Sphingosine 1-phosphate (S1P)/S1P receptors are involved in human liver fibrosis by action on hepatic myofibroblasts motility. *J. Hepatol.* 54 1205–1213. 10.1016/j.jhep.2010.08.028 21145832

[B66] Liu W. Zi M. Tsui H. Chowdhury S. K. Zeef L. Meng Q. J. (2013). A novel immunomodulator, FTY-720 reverses existing cardiac hypertrophy and fibrosis from pressure overload by targeting nfat (nuclear factor of activated t-cells) signaling and periostinclinical perspective. *Circ. Hear. Fail* 6 833–844. 10.1161/CIRCHEARTFAILURE.112.000123 23753531 PMC3871200

[B67] Liu X. Yue S. Li C. Yang L. You H. Li L. (2011). Essential roles of sphingosine 1-phosphate receptor types 1 and 3 in human hepatic stellate cells motility and activation. *J. Cell. Physiol.* 226 2370–2377. 10.1002/jcp.22572 21660960

[B68] Liu Y. Wada R. Yamashita T. Mi Y. Deng C. X. Hobson J. P. (2000). Edg-1, the G protein-coupled receptor for sphingosine-1-phosphate, is essential for vascular maturation. *J. Clin. Invest* 106 951–961. 10.1172/JCI10905 11032855 PMC314347

[B69] Long J. S. Fujiwara Y. Edwards J. Tannahill C. L. Tigyi G. Pyne S. (2010). Sphingosine 1-phosphate receptor 4 uses HER2 (ERBB2) to regulate extracellular signal regulated kinase-1/2 in MDA-MB-453 breast cancer cells. *J. Biol. Chem.* 285 35957–35966. 10.1074/jbc.M110.117945 20837468 PMC2975218

[B70] Lowe N. G. Swaney J. S. Moreno K. M. Sabbadini R. A. (2009). Sphingosine-1-phosphate and sphingosine kinase are critical for transforming growth factor-β-stimulated collagen production by cardiac fibroblasts. *Cardiovasc. Res.* 82 303–312. 10.1093/cvr/cvp056 19228708 PMC2675932

[B71] Lucey M. R. Terrault N. Ojo L. Hay J. E. Neuberger J. Blumberg E. (2013). Long-term management of the successful adult liver transplant: 2013 practice guideline by the American Association for the study of liver diseases and the American Society of Transplantation. *J. Clin. Hepatol.* 19 3–26. 23281277 10.1002/lt.23566

[B72] Maceyka M. Harikumar K. B. Milstien S. Spiegel S. (2012). Sphingosine-1-phosphate signaling and its role in disease. *Trends Cell Biol.* 22 50–60. 10.1016/j.tcb.2011.09.003 22001186 PMC3253987

[B73] Maceyka M. Spiegel S. (2014). Sphingolipid metabolites in inflammatory disease. *Nature* 510 58–67. 10.1038/nature13475 24899305 PMC4320971

[B74] Marques T. G. Chaib E. Da F. J. Lourenço A. C. Silva F. D. Ribeiro Jr (2012). Review of experimental models for inducing hepatic cirrhosis by bile duct ligation and carbon tetrachloride injection. *Acta Cir. Bras.* 27 589–594. 10.1590/S0102-86502012000800013 22850713

[B75] Massberg S. Schaerli P. Knezevic-Maramica I. Köllnberger M. Tubo N. Moseman E. A. (2007). Immunosurveillance by hematopoietic progenitor cells trafficking through blood, lymph, and peripheral tissues. *Cell* 131 994–1008. 10.1016/j.cell.2007.09.047 18045540 PMC2330270

[B76] Means C. K. Xiao C. Y. Li Z. Zhang T. Omens J. H. Ishii I. (2007). Sphingosine 1-phosphate S1P2 and S1P3 receptor-mediated Akt activation protects against *in vivo* myocardial ischemia-reperfusion injury. *Am. J. Physiol. Heart Circ. Physiol.* 292 H2944–H2951. 10.1152/ajpheart.01331.2006 17293497

[B77] Milara J. Navarro R. Juan G. Peiro T. Serrano A. Ramon M. (2012). Sphingosine-1-phosphate is increased in patients with idiopathic pulmonary fibrosis and mediates epithelial to mesenchymal transition. *Thorax* 67 147–156. 10.1136/thoraxjnl-2011-200026 22106015

[B78] Mitra P. Oskeritzian C. A. Payne S. G. Beaven M. A. Milstien S. Spiegel S. (2006). Role of ABCC1 in export of sphingosine-1-phosphate from mast cells. *Proc. Natl. Acad. Sci. U.S.A.* 103 16394–16399. 10.1073/pnas.0603734103 17050692 PMC1637593

[B79] Mizugishi K. Yamashita T. Olivera A. Miller G. F. Spiegel S. Proia R. L. (2005). Essential role for sphingosine kinases in neural and vascular development. *Mol. Cell. Biol.* 25 11113–11121. 10.1128/MCB.25.24.11113-11121.2005 16314531 PMC1316977

[B80] Murata N. Sato K. Kon J. Tomura H. Yanagita M. Kuwabara A. (2000). Interaction of sphingosine 1-phosphate with plasma components, including lipoproteins, regulates the lipid receptor-mediated actions. *Biochem. J.* 352(Pt 3) 809–815. 10.1042/bj3520809 11104690 PMC1221521

[B81] Nagahashi M. Hait N. C. Maceyka M. Avni D. Takabe K. Milstien S. (2014). Sphingosine-1-phosphate in chronic intestinal inflammation and cancer. *Adv. Biol. Regul.* 54 112–120. 10.1016/j.jbior.2013.10.001 24210073 PMC3946530

[B82] Nagahashi M. Kim E. Y. Yamada A. Ramachandran S. Allegood J. C. Hait N. C. (2013). Spns2, a transporter of phosphorylated sphingoid bases, regulates their blood and lymph levels, and the lymphatic network. *FASEB J.* 27 1001–1011. 10.1096/fj.12-219618 23180825 PMC3574288

[B83] Nagahashi M. Yuza K. Hirose Y. Nakajima M. Ramanathan R. Hait N. C. (2016). The roles of bile acids and sphingosine-1-phosphate signaling in the hepatobiliary diseases. *J. Lipid Res.* 57 1636–1643. 10.1194/jlr.R069286 27459945 PMC5003161

[B84] Niederman M. S. Peters S. P. (1998). Update in pulmonary medicine. *Ann. Intern. Med.* 128:208 10.7326/0003-4819-128-3-199802010-00007 9454529

[B85] Nishi T. Kobayashi N. Hisano Y. Kawahara A. Yamaguchi A. (2014). Molecular and physiological functions of sphingosine 1-phosphate transporters. *Biochim. Biophys. Acta* 1841 759–765. 10.1016/j.bbalip.2013.07.012 23921254

[B86] Ohkura S. I. Usui S. Takashima S. I. Takuwa N. Yoshioka K. Okamoto Y. (2017). Augmented sphingosine 1 phosphate receptor-1 signaling in cardiac fibroblasts induces cardiac hypertrophy and fibrosis through angiotensin II and interleukin-6. *PLoS One* 12:e0182329. 10.1371/journal.pone.0182329 28771545 PMC5542600

[B87] Olivera A. Rosenfeldt H. M. Bektas M. Wang F. Ishii I. Chun J. (2003). Sphingosine kinase type 1 induces G12/13-mediated stress fiber formation, yet promotes growth and survival independent of g protein-coupled receptors. *J. Biol. Chem.* 278 46452–46460. 10.1074/jbc.M308749200 12963721

[B88] Opie L. H. Commerford P. J. Gersh B. J. Pfeffer M. A. (2006). Controversies in ventricular remodelling. *Lancet* 367 356–367. 10.1016/S0140-6736(06)68074-4 16443044

[B89] Pan Y. Zhou H. G. Zhou H. Hu M. Tang L. J. (2015). Apolipoprotein M regulates the orphan nuclear receptor LRH-1 gene expression through binding to its promoter region in HepG2 cells. *Drug Des. Dev. Ther.* 9 2375–2382. 10.2147/DDDT.S78496 25987835 PMC4422289

[B90] Park S. J. Im D. S. (2017). Sphingosine 1-phosphate receptor modulators and drug discovery. *Biomol. Ther.* 25 80–90. 10.4062/biomolther.2016.160 PMC5207465 28035084

[B91] Pinzani M. (2002). PDGF and signal transduction in hepatic stellate cells. *Front. Biosci.* 7 d1720–1726. 12133817 10.2741/A875

[B92] Pyne N. J. Dubois G. Pyne S. (2013). Role of sphingosine 1-phosphate and lysophosphatidic acid in fibrosis. *Biochim. Biophys. Acta Mol. Cell Biol. Lipids* 1831 228–238. 10.1016/j.bbalip.2012.07.003 22801038

[B93] Pyne N. J. Pyne S. (2017). Sphingosine 1-phosphate and cancer. *Nat. Rev. Cancer* 10 489–503. 10.1038/nrc2875 20555359

[B94] Pyne S. Adams D. R. Pyne N. J. (2016). Sphingosine 1-phosphate and sphingosine kinases in health and disease: Recent advances. *Prog. Lipid Res.* 62 93–106. 10.1016/j.plipres.2016.03.001 26970273

[B95] Rippe R. A. Brenner D. A. (2004). From quiescence to activation: gene regulation in hepatic stellate cells. *Gastroenterology* 127 1260–1262. 10.1053/j.gastro.2004.08.028 15481004

[B96] Robert P. Tsui P. Laville M. P. Livi G. P. Sarau H. M. Bril A. (2001). EDG1 receptor stimulation leads to cardiac hypertrophy in rat neonatal myocytes. *J. Mol. Cell. Cardiol.* 33 1589–1606. 10.1006/jmcc.2001.1433 11549339

[B97] Russo F. P. Alison M. R. Bigger B. W. Amofah E. Florou A. Amin F. (2006). The bone marrow functionally contributes to liver fibrosis. *Gastroenterology* 130 1807–1821. 10.1053/j.gastro.2006.01.036 16697743

[B98] Ryu Y. Takuwa N. Sugimoto N. Sakurada S. Usui S. Okamoto H. (2002). Sphingosine-1-phosphate, a platelet-derived lysophospholipid mediator, negatively regulates cellular Rac activity and cell migration in vascular smooth muscle cells. *Circ. Res.* 90 325–332. 10.1161/hh0302.104455 11861422

[B99] Salous A. K. Panchatcharam M. Sunkara M. Mueller P. Dong A. Wang Y. (2013). Mechanism of rapid elimination of lysophosphatidic acid and related lipids from the circulation of mice. *J. Lipid Res.* 54 2775–2784. 10.1194/jlr.M039685 23948545 PMC3770090

[B100] Sanchez T. Estrada-Hernandez T. Paik J. H. Wu M. T. Venkataraman K. Brinkmann V. (2003). Phosphorylation and action of the immunomodulator FTY720 inhibits vascular endothelial cell growth factor-induced vascular permeability. *J. Biol. Chem.* 278 47281–47290. 10.1074/jbc.M306896200 12954648

[B101] Sanchez T. Hla T. (2004). Structural and functional characteristics of S1P receptors. *J. Cell. Biochem.* 92 913–922. 10.1002/jcb.20127 15258915

[B102] Sato K. Malchinkhuu E. Horiuchi Y. Mogi C. Tomura H. Tosaka M. (2007). Critical role of ABCA1 transporter in sphingosine 1-phosphate release from astrocytes. *J. Neurochem.* 103 2610–2619. 10.1111/j.1471-4159.2007.04958.x 17931360

[B103] Sato M. Ikeda H. Uranbileg B. Kurano M. Saigusa D. Aoki J. (2016). Sphingosine kinase-1, S1P transporter spinster homolog 2 and S1P2 mRNA expressions are increased in liver with advanced fibrosis in human. *Sci. Rep.* 6:32119. 10.1038/srep32119 27562371 PMC4999825

[B104] Sauer B. Vogler R. Von W. H. Fujii M. Anzano M. B. Glick A. B. (2004). Involvement of Smad signaling in sphingosine 1-phosphate-mediated biological responses of keratinocytes. *J. Biol. Chem.* 279 38471–38479. 10.1074/jbc.M313557200 15247277

[B105] Scarpa M. C. Baraldo S. Marian E. Turato G. Calabrese F. Saetta M. (2013). Ceramide expression and cell homeostasis in chronic obstructive pulmonary disease. *Respiration* 85 342–349. 10.1159/000341185 23018286

[B106] Schwalm S. Pfeilschifter J. Huwiler A. (2013). Sphingosine-1-phosphate: a Janus-faced mediator of fibrotic diseases. *Biochim. Biophys. Acta Mol. Cell Biol. Lipids* 1831 239–250. 10.1016/j.bbalip.2012.07.022 22889995

[B107] Sekiguchi K. Yokoyama T. Kurabayashi M. Okajima F. Nagai R. (1999). Sphingosylphosphorylcholine induces a hypertrophic growth response through the mitogen-activated protein kinase signaling cascade in rat neonatal cardiac myocytes. *Circ. Res.* 85 1000–1008. 10.1161/01.RES.85.11.1000 10571530

[B108] Selman M. King T. E. Pardo A. (2001). Idiopathic pulmonary fibrosis: prevailing and evolving hypotheses about its pathogenesis and implications for therapy. *Ann. Intern. Med.* 134 136–151. 10.7326/0003-4819-134-2-200101160-00015 11177318

[B109] Serra M. Saba J. D. (2010). Sphingosine 1-phosphate lyase, a key regulator of sphingosine 1-phosphate signaling and function. *Adv. Enzyme Regul.* 50 349–362. 10.1016/j.advenzreg.2009.10.024 19914275 PMC2862839

[B110] Shea B. S. Brooks S. F. Fontaine B. A. Chun J. Luster A. D. Tager A. M. (2010). Prolonged exposure to sphingosine 1-phosphate receptor-1 agonists exacerbates vascular leak, fibrosis, and mortality after lung injury. *Am. J. Respir. Cell Mol. Biol.* 43 662–673. 10.1165/rcmb.2009-0345OC 20081052 PMC2993087

[B111] Siwik D. A. Chang D. L. Colucci W. S. (2000). Interleukin-1beta and tumor necrosis factor-alpha decrease collagen synthesis and increase matrix metalloproteinase activity in cardiac fibroblasts *in vitro*. *Circ. Res.* 86 1259–1265. 10.1161/01.RES.86.12.1259 10864917

[B112] Sobel K. Menyhart K. Killer N. Renault B. Bauer Y. Studer R. (2013). Sphingosine 1-phosphate (S1P) receptor agonists mediate pro-fibrotic responses in normal human lung fibroblasts via S1P2 and S1P3 receptors and Smad-independent signaling. *J. Biol. Chem.* 288 14839–14851. 10.1074/jbc.M112.426726 23589284 PMC3663507

[B113] Spiegel S. Milstien S. (2003). Sphingosine-1-phosphate: an enigmatic signalling lipid. *Nat. Rev. Mol. Cell Biol.* 4 397–407. 10.1038/nrm1103 12728273

[B114] Spiegel S. Milstien S. (2007). Functions of the multifaceted family of sphingosine kinases and some close relatives. *J. Biol. Chem.* 282 2125–2129. 10.1074/jbc.R600028200 17135245

[B115] Stanford J. C. Morris A. J. Sunkara M. Popa G. J. Larson K. L. Özcan S. (2012). Sphingosine 1-phosphate (S1P) regulates glucose-stimulated insulin secretion in pancreatic beta cells. *J. Biol. Chem.* 287 13457–13464. 10.1074/jbc.M111.268185 22389505 PMC3339968

[B116] Swaney J. S. Moreno K. M. Gentile A. M. Sabbadini R. A. Stoller G. L. (2008). Sphingosine-1-phosphate (S1P) is a novel fibrotic mediator in the eye. *Exp. Eye Res.* 87 367–375. 10.1016/j.exer.2008.07.005 18687328

[B117] Taha T. A. Hannun Y. A. Obeid L. M. (2006). Sphingosine kinase: biochemical and cellular regulation and role in disease. *J. Biochem. Mol. Biol.* 39 113–131. 10.5483/BMBRep.2006.39.2.113 16584625

[B118] Takabe K. Kim R. H. Allegood J. C. Mitra P. Ramachandran S. Nagahashi M. (2010). Estradiol induces export of sphingosine 1-phosphate from breast cancer cells via ABCC1 and ABCG2. *J. Biol. Chem.* 285 10477–10486. 10.1074/jbc.M109.064162 20110355 PMC2856255

[B119] Takabe K. Spiegel S. (2014). Export of sphingosine-1-phosphate and cancer progression. *J. Lipid Res.* 55 1839–1846. 10.1194/jlr.R046656 24474820 PMC4617347

[B120] Takuwa N. Ohkura S. I. Takashima S. I. Ohtani K. Okamoto Y. Tanaka T. (2010). S1P 3-mediated cardiac fibrosis in sphingosine kinase 1 transgenic mice involves reactive oxygen species. *Cardiovasc. Res.* 85 484–493. 10.1093/cvr/cvp312 19755413 PMC2802201

[B121] Tatler A. L. Jenkins G. (2015). Sphingosine-1-phosphate metabolism: can its enigmatic lyase promote the autophagy of fibrosis? *Thorax* 70 1106–1107. 26530084 10.1136/thoraxjnl-2015-207974

[B122] Thuy A. V. Reimann C. M. Hemdan N. Y. Gräler M. H. (2014). Sphingosine 1-phosphate in blood: function, metabolism, and fate. *Cell. Physiol. Biochem.* 34 158–171. 10.1159/000362992 24977489

[B123] Tsoutsou P. G. Koukourakis M. I. (2006). Radiation pneumonitis and fibrosis: mechanisms underlying its pathogenesis and implications for future research. *Int. J. Radiat. Oncol. Biol. Phys.* 66 1281–1293. 10.1016/j.ijrobp.2006.08.058 17126203

[B124] Urata Y. Nishimura Y. Hirase T. Yokoyama M. (2005). Sphingosine 1-phosphate induces alpha-smooth muscle actin expression in lung fibroblasts via Rho-kinase. *Kobe J. Med. Sci.* 51 17–27. 16199931

[B125] Varga J. Abraham D. (2007). Systemic sclerosis: a prototypic multisystem fibrotic disorder. *J. Clin. Invest.* 117 557–567. 10.1172/JCI31139 17332883 PMC1804347

[B126] Venkataraman K. Thangada S. Michaud J. Oo M. L. Ai Y. Lee Y. M. (2006). Extracellular export of sphingosine kinase-1a contributes to the vascular S1P gradient. *Biochem. J.* 397 461–471. 10.1042/BJ20060251 16623665 PMC1533315

[B127] Wang L. Xing X. P. Holmes A. Wadham C. Gamble J. R. Vadas M. A. (2005). Activation of the sphingosine kinase-signaling pathway by high glucose mediates the proinflammatory phenotype of endothelial cells. *Circ. Res.* 97 891–899. 10.1161/01.RES.0000187469.82595.15 16179586

[B128] Wang R. Ding Q. Yaqoob U. de Assuncao T. M. Verma V. K. Hirsova P. (2015). Exosome adherence and internalization by hepatic stellate cells triggers sphingosine 1-phosphate dependent migration. *J. Biol. Chem.* 290 30684–30696. 10.1074/jbc.M115.671735 26534962 PMC4692200

[B129] Wang Y. Aoki H. Yang J. Peng K. Liu R. Li X. (2017). The role of sphingosine 1-phosphate receptor 2 in bile-acid-induced cholangiocyte proliferation and cholestasis-induced liver injury in mice. *Hepatology* 65 2005–2018. 10.1002/hep.29076 28120434 PMC5444993

[B130] Watterson K. R. Lanning D. A. Diegelmann R. F. Spiegel S. (2007). Regulation of fibroblast functions by lysophospholipid mediators: potential roles in wound healing. *Wound Repair Regen.* 15 607–616. 10.1111/j.1524-475X.2007.00292.x 17971005

[B131] Weichand B. Weis N. Weigert A. Grossmann N. Levkau B. Brüne B. (2013). Apoptotic cells enhance sphingosine-1-phosphate receptor 1 dependent macrophage migration. *Eur. J. Immunol.* 43 3306–3313. 10.1002/eji.201343441 23934754

[B132] Wilkerson B. A. Grass G. D. Wing S. B. Argraves W. S. Argraves K. M. (2012). Sphingosine 1-phosphate (S1P) carrier-dependent regulation of endothelial barrier: high density lipoprotein (HDL)-S1P prolongs endothelial barrier enhancement as compared with albumin-S1P via effects on levels, trafficking, and signaling of S1P1. *J. Biol. Chem.* 287 44645–44653. 10.1074/jbc.M112.423426 23135269 PMC3531779

[B133] Wolters P. J. Collard H. R. Jones K. D. (2014). Pathogenesis of idiopathic pulmonary fibrosis. *Annu. Rev. Pathol.* 9 157–179. 10.1146/annurev-pathol-012513-104706 24050627 PMC4116429

[B134] Woodhoo A. Iruarrizaga-Lejarreta M. Beraza N. García-Rodríguez J. L. Embade N. Fernández-Ramos D. (2012). Human antigen R contributes to hepatic stellate cell activation and liver fibrosis. *Hepatology* 56 1870–1882. 10.1002/hep.25828 22576182 PMC3433583

[B135] Wynn T. A. (2011). Integrating mechanisms of pulmonary fibrosis. *J. Exp. Med.* 208 1339–1350. 10.1084/jem.20110551 21727191 PMC3136685

[B136] Wynn T. A. Ramalingam T. R. (2012). Mechanisms of fibrosis: therapeutic translation for fibrotic disease. *Nat. Med.* 18 1028–1040. 10.1038/nm.2807 22772564 PMC3405917

[B137] Xiu L. Chang N. Yang L. Liu X. Yang L. Ge J. (2015). Intracellular sphingosine 1-phosphate contributes to collagen expression of hepatic myofibroblasts in human liver fibrosis independent of its receptors. *Am. J. Pathol.* 185 387–398. 10.1016/j.ajpath.2014.09.023 25432063

[B138] Yaghobian D. Don A. S. Yaghobian S. Chen X. Pollock C. A. Saad S. (2016). Increased sphingosine 1-phosphate mediates inflammation and fibrosis in tubular injury in diabetic nephropathy. *Clin. Exp. Pharmacol. Physiol.* 43 56–66. 10.1111/1440-1681.12494 26414003

[B139] Yamanaka M. Shegogue D. Pei H. Bu S. Bielawska A. Bielawski J. (2004). Sphingosine kinase 1 (SPHK1) is induced by transforming growth factor-β and mediates timp-1 up-regulation. *J. Biol. Chem.* 279 53994–54001. 10.1074/jbc.M410144200 15485866

[B140] Yanagida K. Hla T. (2016). Vascular and immunobiology of the circulatory sphingosine 1-phosphate gradient. *Annu. Rev. Physiol.* 79 67–91. 10.1146/annurev-physiol-021014-071635 27813829 PMC5500220

[B141] Yang L. Chang N. Liu X. Han Z. Zhu T. Li C. (2012). Bone marrow-derived mesenchymal stem cells differentiate to hepatic myofibroblasts by transforming growth factor-β1 via sphingosine kinase/sphingosine 1-phosphate (S1P)/S1P receptors axis. *Am. J. Pathol.* 181 85–97. 10.1016/j.ajpath.2012.03.014 22609227

[B142] Yang L. Yue S. Yang L. Liu X. Han Z. Zhang Y. (2013). Sphingosine kinase/sphingosine 1-phosphate (S1P)/S1P receptor axis is involved in liver fibrosis-associated angiogenesis. *J. Hepatol.* 59 114–123. 10.1016/j.jhep.2013.02.021 23466305

[B143] Yatomi Y. Ozaki Y. Ohmori T. Igarashi Y. (2001). Sphingosine 1-phosphate: synthesis and release. *Prostaglandins* 64 107–122. 10.1016/S0090-6980(01)00103-4 11324700

[B144] Zhang C. Y. Yuan W. G. He P. Lei J. H. Wang C. X. (2016). Liver fibrosis and hepatic stellate cells: Etiology, pathological hallmarks and therapeutic targets. *World J. Gastroenterol.* 22 10512–10522. 10.3748/wjg.v22.i48.10512 28082803 PMC5192262

[B145] Zhang J. Honbo N. Goetzl E. J. Chatterjee K. Karliner J. S. Gray M. O. (2007). Signals from type 1 sphingosine 1-phosphate receptors enhance adult mouse cardiac myocyte survival during hypoxia. *Am. J. Physiol. Hear. Circ. Physiol.* 293 H3150–H3158. 10.1152/ajpheart.00587.2006 17766476

[B146] Zhao C. G. He X. J. Lu B. Li H. P. Kang A. J. (2010). Increased expression of collagens, transforming growth factor-β1, and -β3 in gluteal muscle contracture. *BMC Musculoskelet. Disord.* 11:15. 10.3109/03008207.2014.964400 20100316 PMC2828420

[B147] Zhao C. G. Qin J. He X. J. Guan Y. C. Jia Y. Lei W. (2013). Sphingosine-1-phosphate is a possible fibrogenic factor in gluteal muscle fibrosis. *Physiol. Res.* 62 691–699. 23869887 10.33549/physiolres.932441

[B148] Zhao Y. Gorshkova I. A. Berdyshev E. He D. Fu P. Ma W. (2011). Protection of LPS-induced murine acute lung injury by sphingosine-1-phosphate lyase suppression. *Am. J. Respir. Cell Mol. Biol.* 45 426–435. 10.1165/rcmb.2010-0422OC 21148740 PMC3175568

[B149] Zhao Y. Natarajan V. (2013). Lysophosphatidic acid (LPA) and its receptors: role in airway inflammation and remodeling. *Biochim. Biophys. Acta Mol. Cell Biol. Lipids* 1831 86–92. 10.1016/j.bbalip.2012.06.014 22809994 PMC3491109

