# Corrigendum: The Role of S1P and the Related Signaling Pathway in the Development of Tissue Fibrosis

**DOI:** 10.3389/fphar.2020.594471

**Published:** 2020-10-30

**Authors:** Erjin Wang, Xingxuan He, Ming Zeng

**Affiliations:** ^1^ Department of Health Toxicology, Xiangya School of Public Health, Central South University, Changsha, China; ^2^ Department of Human Genetics and Genomic Sciences, Icahn Institute for Genomics and Multiscale Biology, Icahn School of Medicine at Mount Sinai, New York, NY, United States

**Keywords:** sphingosine 1-phosphate, tissue fibrosis, SphK, S1PRs, clinical treatment

In the original article, the reference for “Borel et al., 2010” was incorrectly written as “Borel, J. F., Feurer, C., Hiestand, P. C., and Stähelin, H. (2010). Transforming growth factor-beta1 induces transdifferentiation of myoblasts into myofibroblasts *via* up-regulation of sphingosine kinase-1/S1P3 axis. *Mol. Biol. Cell* 21, 1111–1124. doi: 10.1091/mbc.E09-09-0812.” It should be “Cencetti, F., Bernacchioni, C., Nincheri, P., Donati, C., and Bruni, P. (2010). Transforming growth factor-beta1 induces transdifferentiation of myoblasts into myofibroblasts *via* up-regulation of sphingosine kinase-1/S1P3 axis. *Mol. Biol. Cell* 21, 1111–1124. doi: 10.1091/mbc.E09-09-0812.”

The authors apologize for this error and state that this does not change the scientific conclusions of the article in any way. The original article has been updated.

